# Estimation of the Whitefly *Bemisia tabaci* Genome Size Based on k-mer and Flow Cytometric Analyses

**DOI:** 10.3390/insects6030704

**Published:** 2015-07-28

**Authors:** Wenbo Chen, Daniel K. Hasegawa, Kathiravetpillai Arumuganathan, Alvin M. Simmons, William M. Wintermantel, Zhangjun Fei, Kai-Shu Ling

**Affiliations:** 1 Boyce Thompson Institute for Plant Research, Cornell University, Ithaca, NY 14853, USA; E-Mails: wc529@cornell.edu (W.C.); dh574@cornell.edu (D.K.H.); 2 USDA-Agricultural Research Service, U.S. Vegetable Laboratory, Charleston, SC 29414, USA; E-Mail: alvin.simmons@ars.usda.gov; 3 Benaroya Research Institute at Virginia Mason, Seattle, WA 98101, USA; E-Mail: karu@benaroyaresearch.org; 4 USDA-Agricultural Research Service, Salinas, CA 93905, USA; E-Mail: Bill.wintermantel@ars.usda.gov; 5 USDA-Agricultural Research Service, Robert W. Holley Center for Agriculture and Health, Ithaca, NY 14853, USA

**Keywords:** *Bemisia tabaci*, next-generation sequencing, flow cytometry, k-mer analysis

## Abstract

Whiteflies of the *Bemisia tabaci* (Hemiptera: Aleyrodidae) cryptic species complex are among the most important agricultural insect pests in the world. These phloem-feeding insects can colonize over 1000 species of plants worldwide and inflict severe economic losses to crops, mainly through the transmission of pathogenic viruses. Surprisingly, there is very little genomic information about whiteflies. As a starting point to genome sequencing, we report a new estimation of the genome size of the *B. tabaci* B biotype or Middle East-Asia Minor 1 (MEAM1) population. Using an isogenic whitefly colony with over 6500 haploid male individuals for genomic DNA, three paired-end genomic libraries with insert sizes of ~300 bp, 500 bp and 1 Kb were constructed and sequenced on an Illumina HiSeq 2500 system. A total of ~50 billion base pairs of sequences were obtained from each library. K-mer analysis using these sequences revealed that the genome size of the whitefly was ~682.3 Mb. In addition, the flow cytometric analysis estimated the haploid genome size of the whitefly to be ~690 Mb. Considering the congruency between both estimation methods, we predict the haploid genome size of *B. tabaci* MEAM1 to be ~680–690 Mb. Our data provide a baseline for ongoing efforts to assemble and annotate the *B. tabaci* genome.

## 1. Introduction

Whiteflies are among the most important insect pests in the world, causing damage to agricultural, horticultural, and ornamental plants. However, among the over 1500 species of whiteflies that have been reported [1], *Bemisia tabaci* (Gennadius) stands out by exhibiting a remarkable degree of plasticity in host range (over 1000 species) [2], environmental adaptation, insecticide resistance, fecundity, and ability to disperse, while attracting the attention of the agricultural community and scientists worldwide [3].

*B. tabaci* is responsible for transmitting numerous plant pathogenic viruses, most belonging to the genus *Begomovirus* (Family: *Gemniviridae*), as well as other genera such as *Carlavirus*, *Crinivirus*, *Torradovirus*, and *Ipomovirus* [4,5,6,7,8,9,10]. Begomoviruses are transmitted by whiteflies in a persistent manner, and are circulative, *i.e.*, following acquisition, the virus translocates from the gut, into the hemolymph, and eventually into the salivary glands where the virus is egested [11]. For some viruses, protection and circulation in the whitefly is mediated, in part, by secreted factors from host endosymbionts [12,13,14,15,16]. Along with historical evidence, these intimate whitefly-Begomovirus relationships have a rich history together and have presumably been co-evolving for millions of years [11,16]. Aside from understanding the fundamental genetics driving whitefly-endosymbiont-virus relationships, there is a significant importance for developing genetic and genomic resources for managing whiteflies and the viruses they transmit.

*B. tabaci* (Hemiptera: Aleyrodidae) are closely related to aphids, mealybugs, psyllids, and scale insects. Their body sizes range from 2–3 mm in length and, over the course of a lifetime, transition through four instars before the adult stage. Under optimal conditions, *B. tabaci* can undergo 11–15 generations per year and a single female can lay between 100 to 300 eggs during its 3–6 week lifespan [17,18,19]. Like other members of the ancient Aleyrodidae family, *B. tabaci* employs a haplodiploid sex determination system, in which fertilized eggs yield diploid females and unfertilized eggs yield haploid males [20,21]. Considering the nutritional limitations in plant sap, whiteflies, like aphids and many other hemipterans, harbor species-specific mutualistic endosymbionts, which help compensate for deficiencies in certain amino acids [22,23,24,25] and to contribute to the dynamics that have defined the plasticity of *B. tabaci* [14,26,27,28].

*B. tabaci* is a complex of at least 15 cryptic species, or “biotypes” that exhibit a wide range of genetic and phenotypic variations, including differences in virus vectoring potential, host preference and specificity, endosymbiont composition, resistance to insecticides, and reproductive incompatibility [29,30,31]. Here, using flow cytometry and k-mer analysis, we present a new estimate of the genome size for *B. tabaci* Middle East-Asia Minor 1 (MEAM1 [32], also referred to as B biotype), one of the most common and broadly distributed whiteflies in the world.

Flow cytometry has been widely adapted for genome size estimation [33]; although the method is fairly straightforward, the accuracy of estimation is highly dependent on the internal standard and quality of the material used for DNA content measurement [34]. With the rapid development of next generation sequencing, k-mer analysis has recently been developed as an alternative means of genome size estimation [35]. Using both flow cytometry and k-mer analysis, we estimate the *B. tabaci* genome size to be ~680–690 Mb, an estimation that is ~300 Mb smaller than that reported previously using the flow cytometry method alone [36]. Considering our sample material was obtained from whiteflies collected in North America from an isogenic colony, these studies complement and build upon a recent study that estimated the genome size of *B. tabaci* populations in China to be ~640–682 Mb [37]. This updated genome size estimate will serve as the baseline for ongoing genome assembly and annotation efforts.

## 2. Results

**Figure 1:**
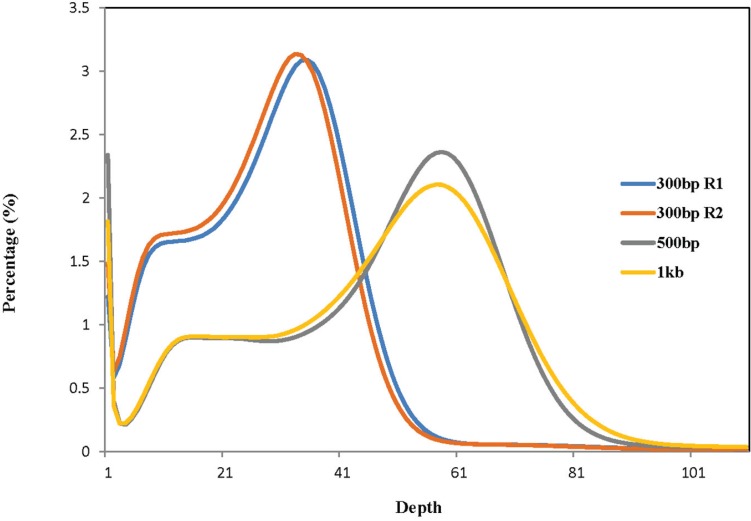
Distribution of unique k-mer depth. The depth of k-mers (size of 27) was plotted against the frequency at which they occurred. Unique k-mers were identified from left (R1) and right (R2) paired-end reads from the library with insert size of ~300 bp, and from all paired-end reads from libraries with insert sizes of 500 bp and 1 Kb, respectively. The peak of the k-mer depth distribution was 35, 33, 58 and 57, respectively. The left smaller peaks indicated a certain degree of heterogeneity of the materials used for genome sequencing of *B. tabaci* MEAM1.

To estimate the genome size of *B. tabaci* using the k-mer approach, we first generated genome sequences using the Illumina deep sequencing technology. Genomic DNA extracted from haploid male individuals derived from an isogenic colony of *B. tabaci* MEAM1 was used to construct three paired-end libraries with insert sizes of approximately 300 bp, 500 bp and 1 Kb, respectively. Each library was sequenced in paired-end mode with read length of 150 bp. A total of approximately 50 billion base pairs of high-quality cleaned sequences were obtained for each of the three libraries, providing sufficient data for a robust k-mer analysis (Table 1). Different k-mer sizes (17, 25, 27 and 99) were tested and all gave nearly identical results for genome size estimation. In this study, results derived from k-mer size of 27 (27-mer) were presented. Left (R1) and right (R2) paired-end reads from the library with insert sizes of ~300 bp were analyzed separately to avoid duplicate counting of 27-mers in the overlap regions of the read pairs. Unique 27-mers were identified in each library from these sequences and the frequency of their occurrences (depths) was derived (Figure 1). The 27-mer depth distributions showed a minor curve (peak) at the left side, indicating a low level of possible heterogeneity within the male individuals collected for sequencing. Based on our k-mer analysis, the genome size of *B. tabaci* MEAM1 was estimated to be 666.7 Mb, 679.2 Mb, 681.4 Mb and 702.3 Mb, respectively, giving an average size of 682.3 Mb (Table 1).

**Table 1:** *Bemisia tabaci* Middle East-Asia Minor 1 (MEAM1) genome size estimation by k-mer analysis.

Library	Total High-Quality Cleaned Bases	Total Number of Corrected 27-mers	Peak Value of 27-mer Depth	Estimated Genome Size (bp) ^b^
300 bp R1 ^a^	28,576,003,381	23,334,888,827	35	666,711,109
300 bp R2 ^a^	27,654,048,868	22,412,934,314	33	679,179,828
500 bp	48,793,881,921	39,521,025,705	58	681,396,995
1 Kb	49,456,289,287	40,032,313,271	57	702,321,285

^a^ R1: left paired-end reads; R2: right paired-end reads. ^b^ Estimated genome size (bp) = total number of k-mer/peak value of k-mer depth distribution.

**Figure 2:**
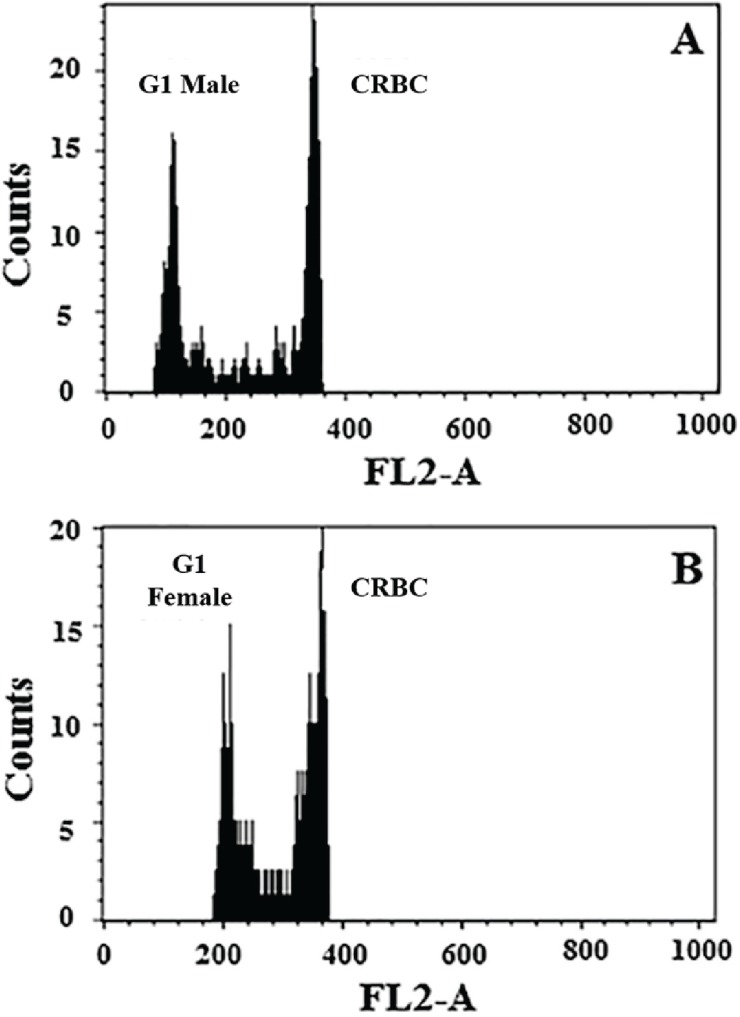
Flow cytometric estimation of the nuclear DNA content of haploid male and diploid female *B. tabaci* MEAM1. Histograms represent relative fluorescence of stained nuclei of male (**A**) and female (**B**) whiteflies relative to the internal standard, chicken red blood cells (CRBC). X-axis = relative nuclear DNA content; Y-axis = number of nuclei.

We subsequently determined the nuclear DNA content of both haploid male and diploid female whiteflies from our isogenic colony using flow cytometry. The analysis was performed in four replications for both males and females. The nuclear DNA content of haploid males was estimated to be 0.73 pg, 0.70 pg, 0.71 pg and 0.69 pg, respectively, and that of diploid females was estimated to be 1.38 pg, 1.38 pg, 1.44 pg and 1.44 pg, respectively, using chicken red blood cells (CRBCs) as the internal standard for comparison (2C = 2.5 pg) (Table 2). As expected, the nuclear DNA content of diploid females was approximately twice that of haploid males (Table 2). Using the conversion of 1 pg DNA = 980 Mb, the haploid genome size of the male whitefly was estimated to be 715.4 Mb, 686 Mb, 695.8 Mb, and 676.2 Mb, respectively, with an average of 693.4 Mb. The haploid genome size of the diploid female whitefly was estimated to be 676.2 Mb, 676.2 Mb, 705.6 Mb and 705.6 Mb, respectively, with an average of 690.9 Mb. Our results demonstrate that the estimated genome sizes of whiteflies using flow cytometry were highly reproducible. Furthermore, histograms produced distinct peaks for both male and female samples, as well as for the CRBC standards, providing confidence in our measurements (Figure 2). Together, our estimates using k-mer and flow cytometric analysis were highly consistent and suggested the haploid genome size of *B. tabaci* MEAM1 to be around 680–690 Mb.

**Table 2:** Flow cytometric estimation of nuclear DNA content of *Bemisia tabaci* MEAM1*.*

	Replicate	Sample	Standard ^a^	DNA Content (pg)
Male (haploid)	1	100.80	343.51	0.73
	2	103.20	366.74	0.70
	3	112.17	396.05	0.71
	4	116.46	419.92	0.69
	Mean ± SD			0.7075 ± 0.0171
	Coefficient of variation			0.024
Female (diploid)	1	190.21	343.96	1.38
	2	205.07	370.81	1.38
	3	230.07	399.54	1.44
	4	243.95	423.25	1.44
	Mean ± SD			1.41 ± 0.0346
	Coefficient of variation			0.025

^a^ Sample and Standard values represent the mean of G0 + G1, with chicken red blood cells used as the internal standard (2.5 pg/2C).

## 3. Discussion

Despite its international notoriety as an insect pest and vector of plant pathogenic viruses, little is known about the genome of the whitefly *Bemisia tabaci*. Accurate determination of the whitefly genome size would be an essential starting point for downstream genomic and genetic analyses. Estimation of genome sizes using the k-mer analysis has been recently applied in various genome sequencing projects [35]. Our k-mer analysis using deep genome sequencing data from three different Illumina libraries produced a genome size estimate of around 680 Mb for *B. tabaci* MEAM1 and flow cytometric analysis of approximately 690 Mb. Together, these data provide robust evidence that the haploid genome size of *B. tabaci* is ~680–690 Mb, a value that is ~300 Mb smaller than that reported previously [36]. We speculate that this discrepancy is largely due to the improvements in the estimation of commonly used standards [33,34] as well as the materials used. In the previous report [36], the chicken red blood cells used as a size standard for estimation of the whitefly genomic DNA content was based on 2C = 3.0 pg DNA. The internal size standard for chicken red blood cells used in the current flow cytometric analysis was based on 2C = 2.5 pg DNA, which was used in the original flow cytometry study on genome estimation of many plant species [33,34].

Whiteflies used in the present study were obtained from a colony that was established from a single female *B. tabaci* MEAM1 collected in the United States in 2013. The use of samples from this isogenic colony was important because it reduced the amount of heterogeneity among whiteflies (as shown in Figure 1), which will also contribute to the assembly and annotation process of ongoing genome sequencing efforts. Our genome size estimate for *B. tabaci* MEAM1 from North America was also confirmed recently by a different team using a Chinese colony of whiteflies (B and Q biotypes) [37]. Together, using whiteflies from North America and China, these two independent studies have provided strong evidence for a new estimated genome size for *B. tabaci* MEAM1. This new genome size is still relatively large in comparison to that of other insects, including fruit fly (*Drosophila melanogaster*) (180 Mb), mosquito (*Anopheles gambiae*) (278 Mb), pea aphid (*Acyrthosiphon pisum*) (464 Mb), and silkworm (*Bombyx mori*) (530 Mb) [38,39,40,41].

Examining insects that have undergone extreme reduction or expansion in genome size, such as the polar-inhabiting Antarctic midge (*Belgica antarctica*) (99 Mb) [42] and the migratory locust (*Locusta migratoria*) (6500 Mb) [43], we see dramatic changes in genomic organization. In the case of the Antarctic midge, analysis revealed that a reduced genome was predominantly attained not through the loss of protein-encoding genes, but rather through reductions in intron length and reduced numbers of repeat elements [42]. Similarly for the migratory locust*,* although the expansion of locus-specific gene families contributed to a large genome size, the majority of the expansion was due to the proliferation of repeat elements and an increased length of intronic regions [43]. In light of these examples of insects that have experienced radical genomic change, it is thought that although the *B. tabaci* genome is nearly four times larger than that of the fruit fly*,* it is anticipated that *B. tabaci* possesses a similar number of genes, and expansions likely occurred in highly repetitive intergenic and intronic regions [36]. Considering the biological novelty of *B. tabaci—*as a complex of cryptic species, having a rich co-evolutionary history with Begomoviruses and other whitefly transmitted viruses, it will be interesting to uncover the underlying factors that have shaped the size and sequence of the *B. tabaci* genome. Together, the data presented herein, with our ongoing efforts to sequence and annotate the whitefly genome, will contribute to these very fundamental understandings.

## 4. Materials and Methods

### 4.1. Insect Rearing

An isogenic whitefly colony was established from a single female *B. tabaci* MEAM1 (B Biotype) collected from a parent colony in April, 2013 at the USDA-ARS, U.S. Vegetable Laboratory in Charleston, South Carolina, USA. The resulting isogenic population was maintained on caged collards (*Brassica oleracea* ssp. acephala de Condolle) in a greenhouse (26 °C ± 5 °C). The MEAM1 population was confirmed by PCR using established primers that amplify the mitochondrial cytochrome oxidase 1 gene [44]. The original source of the parent whitefly colony originated from a collection of adult *B. tabaci* from a sweetpotato (*Ipomea batatas*) field at the U.S. Vegetable Laboratory in 2011. The whiteflies in the parent colony were reared on collard, squash (*Cucurbita pepo*) and tomato (*Solanum lycopersicum*).

### 4.2. DNA Extraction from Male Whiteflies

Whiteflies were aspirated from collards and immediately frozen at −80 °C for 24 h. The whiteflies were then transferred to a Petri dish embedded in a block of ice and using a stereoscope, over 6500 male individuals were collected, pooled and immediately homogenized with a micropestle in the ice-cold lysis buffer (10 mM Tris-EDTA, 0.1 M EDTA pH 8.0, 0.5% *w*/*v* SDS, 1% β-mercaptoethanol). The homogenate was treated with RNase A (Qiagen, Valencia, CA, USA) for 1 h at 37 °C, followed by proteinase K (New England Biolabs, Ipswich, MA, USA) for 2 h at 50 °C. DNA was extracted using phenol:chloroform:isoamyl alcohol (25:24:1). The DNA was ethanol precipitated and rehydrated in TE (pH 8.0) overnight at 4 °C. DNA integrity was analyzed via gel electrophoresis and its quantity was determined on a NanoDrop spectrophotometer (Thermo Fisher Scientific, Waltham, MA, USA).

### 4.3. Genome Size Estimation by k-mer Analysis

Three paired-end libraries with insert sizes of approximately 300 bp, 500 bp and 1 Kb, respectively, were constructed using the Illumina TruSeq DNA sample preparation kit following manufacturer’s instructions, and each library was sequenced on one lane of the Illumina HiSeq 2500 system with the paired-end 150-bp mode at the Roy J. Carver Biotechnology Center, University of Illinois. Raw Illumina reads were processed to remove duplicated read pairs and only one read pair from the duplicates was kept. Duplicated read pairs were defined as those having identical bases in the first 100 bp of both left and right reads. Illumina adaptor and low quality sequences (quality score < 20) were removed from the reads using the ShortRead package [45]. Finally, errors in the Illumina sequencing reads were further corrected using Quake [46].

High-quality cleaned Illumina sequences from each of the three genomic libraries were subjected to k-mer counting using SOAPec (v2.01) in the SOAPdenovo package [47] with the k-mer size set to 27. Because the read length was 150 bp, for the library with insert size of 300 bp, k-mers were counted separately for left and right paired-end reads in order to avoid possible duplicate counting of k-mers in the overlap region of the read pairs. K-mer depth distribution was then derived and the peak value of the depth distribution identified. In shot-gun genome sequencing, the short reads are assumed to be randomly generated, so any k-mers in the reads also occur randomly. Their depth of coverage follows the Poisson distribution [48] and the mean of k-mer depth should be equal to the peak value of the k-mer depth distribution. Thus, genome size can be estimated using the following formula:

Genome size = total number of k-mers/peak value of k-mer frequency distribution.

### 4.4. Nuclear DNA Content Estimation by Flow Cytometry

The procedure used to analyze the nuclear DNA content of the whiteflies was modified from Arumuganathan and Earle [33]. Ten male or female whiteflies fixed in 95% ethanol were chopped vigorously and suspended in a 0.5 mL solution of 10 mM MgSO_4_.7H_2_O, 50 mM KCl, 5 mM HEPES, pH 8.0, 3 mM dithiothreitol, 0.1 mg/mL propidium iodide, 1.5 mg/mL DNase-free RNase and 0.25% Triton X-100. The suspended nuclei were withdrawn using a pipette, filtered through a 30 µm nylon mesh, and incubated at 37 °C for 30 min before flow cytometric analysis. Suspensions of sample nuclei were spiked with a suspension of standard nuclei (prepared in the above solution) and analyzed with a FACScalibur flow cytometer (Becton-Dickinson, San Jose, CA, USA). For each measurement, the propidium iodide fluorescence area signals (FL2-A) from 1000 nuclei were collected and analyzed by the CellQuest software (Becton-Dickinson). The mean position of the G0/G1 (Nuclei) peak of the sample and the internal standard were determined using CellQuest software. The mean nuclear DNA content of each sample, measured in picograms, was based on 1000 scanned nuclei. Nuclei from chicken red blood cells (2.5 pg/2C) [33,34] were used as an internal standard. Each experiment was conducted with four replications for male or female whiteflies. Whitefly nuclear DNA content was derived using the following formula:

Nuclear DNA content = (Mean position of whitefly nuclei peak/Mean position of CRBC nuclei peak) × 2.5 pg.

## 5. Conclusions

In this study, we provide a new genome size estimate for *B. tabaci* (MEAM1), one of the most destructive and invasive whitefly species in the world. Results presented in this study were obtained from a population of *B. tabaci* MEAM1 from North America. Specifically, the insects used in this study were from a population that originated from a field site where this whitefly occurs year-round and feral populations have been known at this location since 1990 (originally reported as *B. argentifolii*) [49]. We collected the haploid male whiteflies derived from a single isoline colony to reduce the level of heterogeneity in the population and utilized flow cytometry and k-mer analysis to conclude that the haploid genome of *B. tabaci* MEAM1 is ~680–690 Mb. Flow cytometry on diploid female whiteflies predicted a genome size approximately twice that of males, further validating our estimations. This new estimation is ~300 Mb smaller than previously reported [36]. Our study provides a starting point for sequencing and annotating the whitefly genome, and will ultimately contribute to our understanding of whitefly biology and the generation of novel pest management strategies.

## References

[1] Martin J.H., Mound L.A. (2007). An annotated check list of the world’s whiteflies (Insecta: Hemiptera: Aleyrodidae). Zootaxa.

[2] Abd-Rabou S., Simmons A.M. (2010). Survey of reproductive host plants of *Bemisia*
*tabaci* (Hemiptera: Aleyrodidae) in Egypt, including new host records. Entomol. News.

[3] Brown J.K. (1994). Current status of *Bemisia tabaci* as a plant pest and virus vector in agroecosystems worldwide. FAO Plant Prot. Bull..

[4] Jones D.R. (2003). Plant viruses transmitted by whiteflies. Eur. J. Plant Pathol..

[5] Legg J.P., Owor B., Sseruwagi P., Ndunguru J. (2006). Cassava mosaic virus disease in East and Central Africa: Epidemiology and management of a regional pandemic. Adv. Virus Res..

[6] Legg J.P., Stansly P.A., Naranjo S.E. (2010). Epidemiology of a whitefly-transmitted cassava mosaic geminivirus pandemic in Africa. Bemisia: Bionomics and Management of a Global Pest.

[7] Diaz-Pendon J.A., Canizares M.C., Moriones E., Bejarano E.R., Czosnek H., Navas-Castillo J. (2010). Tomato yellow leaf curl viruses: Menage a trois between the virus complex, the plant and the whitefly vector. Mol. Plant Pathol..

[8] Hanssen I.M., Lapidot M., Thomma B.P. (2010). Emerging viral diseases of tomato crops. Mol. Plant Microbe Interact..

[9] Navas-Castillo J., Fiallo-Olive E., Sanchez-Campos S. (2011). Emerging virus diseases transmitted by whiteflies. Annu. Rev. Phytopathol..

[10] Van der Vlugt R.A., Verbeek M., Dullemans A.M., Wintermantel W.M., Cuellar W.J., Fox A., Thompson J.R. (2015). Torradoviruses. Annu. Rev. Phytopathol..

[11] Czosnek H., Ghanim M. (2012). Back to basics: Are Begomoviruses whitefly pathogens?. J. Integr. Agric..

[12] Morin S., Ghanim M., Zeidan M., Czosnek H., Verbeek M., van den Heuvel J.F.J.M. (1999). A GroEL homologue from endosymbiotic bacteria of the whitefly *Bemisia tabaci* is implicated in the circulative transmission of *Tomato yellow leaf curl virus*. Virology.

[13] Morin S., Ghanim M., Sobol I., Czosnek H. (2000). The GroEL protein of the whitefly *Bemisia tabaci* interacts with the coat protein of transmissible and nontransmissible begomoviruses in the yeast two-hybrid system. Virology.

[14] Gottlieb Y., Zchori-Fein E., Mozes-Daube N., Kontsedalov S., Skaljac M., Brumin M., Sobol I., Czosnek H., Vavre F., Fleury F. (2010). The transmission efficiency of *Tomato yellow leaf curl virus* by the whitefly *Bemisia tabaci* is correlated with the presence of a specific symbiotic bacterium species. J. Virol..

[15] Kliot A., Ghanim M. (2013). The role of bacterial chaperones in the circulative transmission of plant viruses by insect vectors. Viruses.

[16] Ghanim M. (2014). A review of the mechanisms and components that determine the transmission efficiency of *Tomato yellow leaf curl virus* (Geminiviridae; *Begomovirus*) by its whitefly vector. Virus Res..

[17] Avidov Z. (1956). Bionomics of the tobacco whitefly (*Bemisia tabaci* Gennad.) in Israel. Ktavim.

[18] Azab A.K., Megahed M.M., El-Mirsawi H.D. (1971). On the biology of *Bemisia tabaci* (Genn.) (Hemiptera-Homoptera: Aleyrodidae). Bull. Entomol. Soc. Egypte.

[19] Bethke J.A., Paine T.D., Nuessly G.S. (1991). Comparative biology, morphometrics, and development of two populations of *Bemisia tabaci* (Homoptera: Aleyrodidae) on cotton and poinsettia. Ann. Entomol. Soc. Am..

[20] Schrader F. (1920). Sex determination in the white-fly (*Trialeurodes vaporariorum*). J. Morphol..

[21] Blackman R.L., Cahill M. (1998). The karyotype of *Bemisia tabaci* (Hemiptera: Aleyrodidae). Bull. Entomol. Res..

[22] Costa H.S., Westcot D.M., Ullman D.E., Johnson M.W. (1993). Ultrastructure of the endosymbionts of the whitefly, *Bemisia tabaci* and *Trialeurodes vaporariorum*. Protoplasma.

[23] Costa H.S., Westcot D.M., Ullman D.E., Rosell R., Brown J.B., Johnson M.W. (1995). Morphological variation in *Bemisia* endosymbionts. Protoplasma.

[24] Szklarzewicz T., Moskal A. (2001). Ultrastructure, distribution, and transmission of endosymbionts in the whitefly *Aleurochiton aceris* Modeer (Insecta, Hemiptera, Aleyrodinea). Protoplasma.

[25] Baumann P. (2005). Biology of bacteriocyte-associated endosymbionts of plant sap-sucking insects. Ann. Rev. Microbiol..

[26] Chiel E., Gottlieb Y., Zchori-Fein E., Mozes-Daube N., Katzir N., Inbar M., Ghanim M. (2007). Biotype-dependent secondary symbiont communities in sympatric populations of *Bemisia tabaci*. Bull. Entomol. Res..

[27] Bing X.L., Ruan Y.M., Rao Q., Wang X.W., Liu S.S. (2013). Diversity of secondary endosymbionts among different putative species of the whitefly *Bemisia tabaci*. Insect Sci..

[28] Zchori-Fein E., Lahav T., Freilich S. (2014). Variations in the identity and complexity of endosymbiont combinations in whitefly hosts. Front. Microbiol..

[29] Zang L.S., Chen W.Q., Liu S.S. (2006). Comparison of performance on different host plants between the B biotype and a non-B biotype of *Bemisia tabaci* from Zhejiang, China. Entomol. Exp. Appl..

[30] Houndété T.A., Kétoh G.K., Hema O.S., Brévault T., Glitho I.A., Martin T. (2010). Insecticide resistance in field populations of *Bemisia tabaci* (Hemiptera: Aleyrodidae) in West Africa. Pest Manag. Sci..

[31] Bedford I.D., Briddon R.W., Brown J.K., Roswell R.C., Markham P.G. (1994). Geminivirus transmission and biological characterisation of *Bemisia tabaci* (Gennadius) biotypes from different geographic regions. Ann. Appl. Biol..

[32] Dinsdale A., Cook L., Riginos C., Buckley Y.M., de Barro P.J. (2010). Refined global analysis of *Bemisia tabaci* (Hemiptera: Sternorrhyncha: Aleyrodoidea: Aleyrodidae) mitochondrial cytochrome oxidase 1 to identify species level genetic boundaries. Ann. Entomol. Soc. Am..

[33] Arumuganathan K., Earle E.D. (1991). Nuclear DNA content of some important plant species. Plant Mol. Biol. Rep..

[34] Dolezel J., Greilhuber J. (2010). Nuclear genome size: Are we getting closer?. Cytometry A.

[35] Liu B., Shi Y., Yuan J., Hu X., Zhang H., Li N., Li Z., Chen Y., Mu D., Fan W. Estimation of genomic characteristics by analyzing k-mer frequency in *de novo* genome projects. http://arxiv.org/ftp/arxiv/papers/1308/1308.2012.pdf.

[36] Brown J.K., Lambert G.M., Ghanim M., Czosnek H., Galbraith D.W. (2005). Nuclear DNA content of the whitefly *Bemisia tabaci* (Aleyrodidae: Hemiptera) estimated by flow cytometry. Bull. Entomol. Res..

[37] Guo L., Wang S., Wu Q., Zhou X., Xie W., Zhang Y. (2015). Flow cytometry and K-mer analysis estimates of the genome sizes of *Bemisia tabaci* B and Q (Hemiptera: Aleyrodidae). Front. Physiol..

[38] Adams M.D., Celniker S.E., Holt R.A., Evans C.A., Gocayne J.D., Amanatides P.G., Scherer S.E., Li P.W., Hoskins R.A., Galle R.F. (2000). The genome sequence of *Drosophila melanogaster*. Science.

[39] Holt R.A., Subramanian G.M., Halpern A., Sutton G.G., Charlab R., Nusskern D.R., Wincker P., Clark A.G., Ribeiro J.M., Wides R. (2002). The genome sequence of the malaria mosquito *Anopheles gambiae*. Science.

[40] The International Aphid Genomics Consortium (2010). Genome sequence of the pea aphid *Acyrthosiphon pisum*. PLoS Biol.

[41] Mita K., Kasahara M., Sasaki S., Nagayasu Y., Yamada T., Kanamori H., Namiki N., Kitagawa M., Yamashita H., Yasukochi Y. (2004). The genome sequence of silkworm, *Bombyx mori*. DNA Res..

[42] Kelley J.L., Peyton J.T., Fiston-Lavier A.S., Teets N.M., Yee M.C., Bustamante C.D., Lee R.E., Denlinger D.L. (2014). Compact genome of the Antarctic midge is likely an adaptation to an extreme environment. Nat. Commun..

[43] Wang X., Fang X., Yang P., Jiang X., Jiang F., Zhao D., Li B., Cui F., Wei J., Ma C. (2014). The locust genome provides insight into swarm formation and long-distance flight. Nat. Commun..

[44] Shatters R.G., Powell C.A., Boykin L.M., Liansheng H., McKenzie C.L. (2009). Improved DNA barcoding method for *Bemisia tabaci* and related Aleyrodidae: Development of universal and *Bemisia tabaci* biotype-specific mitochondrial cytochrome c oxidase I polymerase chain reaction primers. J. Econ. Entomol..

[45] Morgan M., Anders S., Lawrence M., Aboyoun P., Pagès H., Gentleman R. (2009). ShortRead: A Bioconductor package for input, quality assessment and exploration of high-throughput sequence data. Bioinformatics.

[46] Kelley D.R., Schatz M.C., Salzberg S.L. (2010). Quake: Quality-aware detection and correction of sequencing errors. Genome Biol..

[47] Luo R., Liu B., Xie Y., Li Z., Huang W., Yuan J., He G., Chen Y., Pan Q., Liu Y. (2012). SOAPdenovo2: An empirically improved memory-efficient short-read *de novo* assembler. GigaScience.

[48] Li X., Waterman M.S. (2003). Estimating the repeat structure and length of DNA sequences using ℓ-tuples. Genome Res..

[49] Simmons A.M., Elsey K.D. (1995). Overwintering and cold tolerance of *Bemisia argentifolii* (Homoptera: Aleyrodidae) in coastal South Carolina. J. Entomol. Sci..

